# Male-Dominant Activation of Rat Renal Organic Anion Transporter 1 (Oat1) and 3 (Oat3) Expression by Transcription Factor BCL6

**DOI:** 10.1371/journal.pone.0035556

**Published:** 2012-04-18

**Authors:** Waja Wegner, Birgitta Christina Burckhardt, Gerhard Burckhardt, Maja Henjakovic

**Affiliations:** Department of Systemic Physiology and Pathophysiology, University Medical Center Göttingen, Göttingen, Germany; University of Geneva, Switzerland

## Abstract

**Background:**

Organic anion transporters 1 (Oat1) and 3 (Oat3) mediate the transport of organic anions, including frequently prescribed drugs, across cell membranes in kidney proximal tubule cells. In rats, these transporters are known to be male-dominant and testosterone-dependently expressed. The molecular mechanisms that are involved in the sex-dependent expression are unknown. Our aim was to identify genes that show a sex-dependent expression and could be involved in male-dominant regulation of Oat1 and Oat3.

**Methodology/Principal Findings:**

Promoter activities of Oat1 and Oat3 were analyzed using luciferase assays. Expression profiling was done using a SurePrint G3 rat GE 8×60K microarray. RNA was isolated from renal cortical slices of four adult rats per sex. To filter the achieved microarray data for genes expressed in proximal tubule cells, transcription database alignment was carried out. We demonstrate that predicted androgen response elements in the promoters of Oat1 and Oat3 are not functional when the promoters were expressed in OK cells. Using microarray analyses we analyzed 17,406 different genes. Out of these genes, 56 exhibit a sex-dependent expression in rat proximal tubule cells. As genes potentially involved in the regulation of Oat1 and Oat3 expression, we identified, amongst others, the male-dominant hydroxysteroid (17-beta) dehydrogenase 1 (Hsd17b1), B-cell CLL/lymphoma 6 (BCL6), and polymerase (RNA) III (DNA directed) polypeptide G (Polr3g). Moreover, our results revealed that the transcription factor BCL6 activates promoter constructs of Oat1 and Oat3.

**Conclusion:**

The results indicate that the male-dominant expression of both transporters, Oat1 and Oat3, is possibly not directly regulated by the classical androgen receptor mediated transcriptional pathway but appears to be regulated by the transcription factor BCL6.

## Introduction

There are several known differences in the mode of drug action between men and women, however the effect of sex-dependent dosages is just beginning to be explored [1]. The reasons for different drug actions are not entirely clear. Sex differences are found in the absorption, distribution, metabolism and elimination of drugs [1]–[3]. The liver and kidneys are responsible for drug elimination. Hepatocytes in the liver and proximal tubule cells in the kidneys express several transport proteins such as members of the ATP-binding cassette (ABC) transporters, e.g. multidrug resistance proteins (MRPs), or members of the solute carrier (SLC) family 22A, e.g. organic anion transporters (OATs) and organic cation transporters (OCTs), that are involved in the excretion of endogenous and exogenous substrates [4]–[6]. Sex differences in the transport of substrates have been shown and could contribute to interindividual variations in drug efficacy [7]. In 2010, “The International Transporter Consortium” published a recommendation as to which of the transport proteins clinically important in drug absorption and elimination have to be analyzed in drug development [8]. Amongst others, the human organic anion transporter 1 (OAT1) and 3 (OAT3) were mentioned as clinically relevant transporters in the kidneys [8]. Immunohistochemical experiments revealed that OAT1 and OAT3 are expressed at the basolateral membrane of proximal tubule kidney cells in humans [9], [10] and rats [11], [12]. OAT1 and OAT3 are responsible for the uptake of their substrates from the blood into the cells and interact with several drugs, e.g. analgesics, antibiotics, and antivirals [5], [13]–[15]. For example, the often consumed analgesic ibuprofen is transported by human OAT1 as well as by OAT3 [16] and causes more adverse drug reactions (ADRs) in women than in men [17]. For a multitude of different drugs, e.g. analgesics, ACE-inhibitors, and antihistamines, women have a 1.6-fold higher risk of getting an ADR compared to men [18]. This phenomenon could be partially due to sex-dependent differences in the expression of transporters like OAT1 and OAT3. A lower expression of OAT1 or OAT3 may decrease drug excretion. Rats that are often used in preclinical trials exhibit sex-dependent differences in Oat1 and Oat3, with a higher expression in males compared to females [19]. In rats, expression of Oat1 and Oat3 is increased by testosterone and decreased by estradiol [19]. The molecular mechanisms of sex-dependent expression of Oat1 and Oat3 are still unclear.

The purpose of this study was to identify genes that demonstrate a sex-dependent expression in rat proximal tubule cells and could be related to male-dominant expression of Oat1 and Oat3. We identified as a promising candidate gene BCL6 that shows a male-dominant expression, and might be involved in the regulation of Oat1 and Oat3.

## Materials and Methods

### Cloning of the 5′-regulatory regions (promoter) of Oat1- and Oat3-genes into pGL3-Enhancer

The transcriptional start site of Oat1 and Oat3 was identified by the alignment of genomic and mRNA sequences from each gene (GenBank accession numbers: Oat1 genomic, NW_047563.2; mRNA, NM_017224.2; Oat3 genomic, NW_047563.2; mRNA, NM_031332.1). Based on rat genomic sequence, promoter fragments of varying lengths were amplified by polymerase chain reaction (PCR) using rat genomic DNA (BioCat, Germany) as templates. Primers shown in Table 1 were designed according to sequences in the 5′-regulatory region. Primer numbering indicated their position relative to the transcriptional start site (designated as +1). Amplified PCR products were digested with their corresponding restriction enzymes (New England BioLabs GmbH, Germany) and ligated into the pGL3-Enhancer vector (Promega, Germany) (Table 1). The full length promoter construct of Oat1 is referred to as −2252/+113, the next shorter one as −1666/+113, and the shortest one as −1226/+113. The promoter constructs for Oat3 are designated as −2567/+12 for the full length one, −752/+12 for the next shorter one, and −444/+12 for the shortest promoter construct.

**Table 1:** Cloning of different promoter constructs of Oat1 and Oat3.

Position	Sequence	Enzyme	Backbone vector
**Oat1 (−1226/+113)**			
−1226 to −1207	5′-*gg* **GCTAGC**AGTGAGATGACAGGCAAAGG-3′	NheI/XhoI	pGL3-Enhancer
+94 to +113	5′-*gg* **CTCGAG**ATGGTGACCTGGATCAACTG-3′		
**Oat1 (−1666/+113)**			
−1666 to −1647	5′-*gg* **GCTAGC**CAGAGTGAGTTCCAGGACAG-3′	NheI/NdeI	Oat1 (−1226/+113)
−1131 to −1111	5′-*gggggg*GCATGGAGGTCTGAGGATAAC-3′		
**Oat1 (−2252/+113)**			
−2252 to −2232	5′-*gg* **GCTAGC**CTGCCCAAGATGATTTTGAAC-3′	NheI/BbvCI	Oat1 (−1666/+113)
−1545 to −1526	5′-*gcgcg*ATTGGAGCTGAGGACTGAAC-3′		
**Oat3 (−444/+12)**			
−444 to −425	5′-*gg* **GAGCTC**CCCTAACATTCTCCACGAAC-3′	SacI/XhoI	pGL3-Enhancer
−7 to +12	5′-*gg* **CTCGAG**AGGACAGCTCAGCTCTAAC-3′		
**Oat3 (−752/+12)**			
−752 to −732	5′-*gg* **GAGCTC**GGAGAACAGACAACCACATAC-3′	SacI/XhoI	pGL3-Enhancer
−7 to +12	5′-*gg* **CTCGAG**AGGACAGCTCAGCTCTAAC-3′		
**Oat3 (−2567/+12)**			
−1823 to −1802	5′-*ggg* **GAGCTC**GGCCAACTAGGAACTAGAAAAG-3′	SacI/AvrII	Oat3 (−752/+12)
−685 to −665	5′-*gcggcggg*CAAGCCAACACAAACTGAATC-3′		
−2631 to −2613	5′-TGAGATGAGGACAACGGAC-3′	SacI/PstI	Oat3 (−1823/+12)
−1702 to −1681	5′-TTAAATTATACTCCAAGGCCGC-3′		

Oat: organic anion transporter; bold nucleotides: artificial restriction sites (not included in numbering); small italic nucleotides: adjustment of primer melting temperature (not included in numbering).

### *In silico* analyses of putative transcription factor binding sites

For determination of predicted transcription factor binding sites, based on genomic sequence, 3 kilobase (kb) upstream of the transcription start site of Oat1 (NW_047563.2) and Oat3 (NW_047563.2) were investigated. The analyses were performed using MatInspector (http://www.genomatix.de/).

### Cell culture, transfection, and luciferase assay

Opossum kidney (OK) cells were purchased from LGC Standards (Germany; European distributor for “ATCC cultures and bioproducts”; ATCC® number: CRL-1840™). OK cells were cultured in Quantum 286 medium (complete medium for epithelial cells with L-glutamine, PAA Laboratories GmbH, Austria) with 100 units/ml penicillin and 100 µg/ml streptomycin (PAA Laboratories GmbH, Austria) at 37°C in a 5% CO_2_-95% air humidified incubator. Cells (0.75×10^5^ cells/well) were seeded into 24-well culture plates in their appropriate culture medium without antibiotics, incubated up to 24 h and then transiently transfected using Lipofectamine™ 2000 (Invitrogen, Germany) according to the manufacturer's recommendations. OK cells were transfected at a confluence of approximately 70% with 0.5 µg promoter construct (Oat1, Oat3) or pGL3-Enhancer (Promega, Germany), 0.5 µg rPb-Luc, the minimal promoter of rat probasin cloned in pGL3-Basic (kind gift from Silke Kaulfuss, Institute of Human Genetics, University of Göttingen, and described in [20]) or pGL3-Basic (Promega, Germany), 0.5 µg of the B-cell CLL/lymphoma 6 (BCL6) expression vector pcDNA3-BCL6 (kind gift from Giovanna Roncador, Monoclonal Antibodies Unit Centro Nacional de Investigaciones Oncológicas, Spain, and described in [21]) or 0.5 µg empty vector pcDNA3, 0.1 µg of the rat androgen receptor (rAR) expression vector pSG5-rAR (kind gift from Olli A. Jänne, Institute of Biomedicine, University of Helsinki, Finland, and described in [22]) or 0.1 µg empty vector pSG5 and 25 ng pRL-TK (*Renilla reniformis* vector, Promega, Germany) as indicated in the figures. Five hours after transfection of OK cells, medium was changed to complete medium with antibiotics in case of BCL6-transfection. For rAR-transfection medium was supplemented with 100 nM testosterone (Fluka, Germany) dissolved in dimethyl sulfoxide (DMSO) (AppliChem, Germany), or control 0.0003% DMSO, in the absence of antibiotics. Incubations were stopped after 43 h and firefly and *Renilla* luciferase activity were determined using the Dual-Luciferase® Reporter Assay System (Promega, Germany). The luminescences were measured using Mithras LB940 luminometer (Berthold, Germany). Firefly luciferase activity was normalized to *Renilla* luciferase activity. Data are presented as the fold increase over pGL3-Basic or pGL3-Enhancer activity.

### Immunofluorescence analysis of BCL6- and rAR-expression

OK cells were seeded (0.75×10^5^ cells/well) on poly-D-lysine (Sigma Aldrich, Germany) coated cover slips into 24-well culture plates. Cells were transiently transfected with either 0.1 µg pSG5-rAR or control pSG5 for rAR-expression or with 0.5 µg pcDNA3-BCL6 or control pcDNA3 for BCL6-expression as described above. 43 h after transfection cells were fixed in 3.7% paraformaldehyde (Merck, Germany) in phosphate buffered saline (PBS) (Applichem, Germany) for 8 min at room temperature (RT), and subsequently permeabilized for 5 min at RT in a buffer consisting of 50 mM Na_2_HPO_4_/NaH_2_PO_4_, pH 7.4; 0.5 mM NaCl, 0.3% Triton X-100 (Carl Roth, Germany). For staining, antibodies were diluted in PBS containing 0.1% bovine serum albumin (BSA) (Carl Roth, Germany). Cells were first incubated for 2 h at RT with 0.5 µg/ml rabbit anti-rat AR polyclonal antibody (Santa Cruz, USA) or 0.5 µg/ml mouse anti-human BCL6 monoclonal antibody (Santa Cruz, USA), blocked with 1% BSA for 15 min at RT, incubated for 1 h at RT with 8 µg/ml Alexa Fluor® 488 labeled goat anti-rabbit IgG (H+L) (Invitrogen, Germany) or 8 µg/ml Alexa Fluor® 488 labeled goat anti-mouse IgG (H+L) (Invitrogen, Germany), and finally incubated for 5 min at RT with 300 nM 4′,6-diamidino-2-phenylindole dihydrochloride (DAPI) (Invitrogen, Germany). Cells were washed with PBS and investigated by fluorescence microscopy. For evaluation of transfection efficiency, cells were investigated under a 20× magnification and five pictures across one cover slip were made. Using Image J (version 1.44, National Institutes of Health, USA), DAPI (blue) stained cells were counted and set as 100%. BCL6- and rAR-positive cells, stained in green, were further counted and their ratio to DAPI stained cells was calculated. The mean of all five pictures was estimated and is referred as transfection efficiency in %.

### Ethic statement

N/A

### Animals

RCCHan™∶WIST rats were obtained from Harlan Laboratories (Venray, Netherlands). Animals were kept in the animal facility of the University Medical Center Göttingen under conventional housing conditions (22°C, 55% humidity, and 12 h day/night cycle) given free access to water and rat chow. Acclimatization period was at least 14 days. Four male and four female rats were anesthetized with CO_2_ and euthanized by cervical dislocation. The kidneys were extracted post mortem in accordance with the guidelines of the “German Animal Welfare Act” (German: Tierschutzgesetz; §4 Abs. 3). We did not need formal approval, because the study did not involve any treatments or harmful procedures. The experiments were notified by animal welfare office of University Medical Center Göttingen, Germany.

### Tissue preparation and RNA isolation

The kidneys were extracted directly post mortem, washed in cold PBS and cooled on ice. After removal of the kidneys capsules, cortical slices were prepared and stored overnight in RNA*later*® (Applied Biosystems, Germany) at 4°C. Total RNA was isolated from kidney cortical slices using RNeasy® Mini Kit (Qiagen, Germany) according to the manufacturer's recommendations.

Quality and quantity of extracted RNA were determined using the Agilent 2100 Bioanalyzer (Agilent Technologies, USA) and NanoDrop® ND-1000 Spectrophotometer (Thermo Scientific NanoDrop Technologies, USA) following the manufacturer's protocol. RNAs with a RNA integrity number (RIN) >9 were used for further experiments.

### Microarray procedure and data analyses

For microarray experiments, total RNA from four male and four female rat cortical kidney slices, each slice as a separate sample, were analyzed. Microarray preparation was performed as described previously by Opitz *et al.* [23] with the following exceptions: 200 ng of total RNA as starting material, the “Low Input Quick Amp Labeling Kit, one-color” (Agilent Technologies, USA), the SurePrint G3 Rat GE 8×60K Kit (Agilent Technologies, USA), and Agilent DNA microarray scanner (G2505C) at 3 micron resolution were used.

Data analyses were also performed as described previously [23] with the exception that Agilent's Feature (FE) software version 10.7.3.1 was used. All microarray data were generated conforming to the Minimum Information About a Microarray Experiment (MIAME) guidelines and have been submitted to the Gene Expression Omnibus (GEO) database at http://www.ncbi.nlm.nih.gov/geo/ (accession number: GSE34565). For evaluation of sex-different expression profiles four adjustments were made: 1) *p*-value<0.05, 2) log2 fold-change (FC)≥1 (higher expressed in male rats) and log2 fold-change (FC)≤−1 (higher expressed in female rats), 3) false discovery rate (FDR) <5%, and 4) duplicated genes were removed and the ones with the lower log2 fold-change were kept. To identify genes that are expressed in the rat proximal tubules candidate genes were aligned with the “Rat Proximal Tubule Transcriptome Database” (http://dir.nhlbi.nih.gov/papers/lkem/pttr/). Volcano plot for sex-dependent gene expression was performed by using plot function in “R” (http://cran.r-project.org/).

### TaqMan® real-time polymerase chain reaction

For gene expression analysis, RNA of the four male and four female rat cortical kidney slices were reverse transcribed using oligo dT-primer (Eurofins MWG Operon, Germany) and Superscript™ II Reverse Transcriptase (Invitrogen, Germany). The detection of genes of interest was realized by TaqMan® Gene Expression Assays (Applied Biosystems, USA). As endogenous control, mRNA expression of housekeeping gene beta-actin (β-actin) or hypoxanthine phosphoribosyltransferase 1 (Hprt1) was monitored. Following TaqMan® Gene Expression Assays were used: β-actin, Rn00667869_m1; Hprt1, Rn01527840_m1; hepatocyte nuclear factor 1α (Hnf1α), Rn00562020_m1; hepatocyte nuclear factor 1β (Hnf1β), Rn00447453_m1; hepatocyte nuclear factor 4α (Hnf4α), Rn00573309_m1; androgen receptor (rAR), Rn00560747_m1; Oat1, Rn00568143_m1; Oat3, Rn00580082_m1; polymerase (RNA) III (DNA directed) polypeptide G (Polr3g), Rn01494192_m1; hydroxysteroid (17-beta) dehydrogenase 1 (Hsd17b1), Rn00563388_g1; B-cell CLL/lymphoma 6 (BCL6), Rn01404339_m1. TaqMan® real-time PCR analysis was performed in a Mx3000P™ real-time PCR cycler (Agilent Technologies, USA) with the following parameters: 2 min heating step at 50°C, 10 min hot start at 95°C, followed by 40 cycles of 95°C for 15 s and 60°C for 1 min. The amplification efficiency of all used TaqMan® Gene Expression Assays was 100% (+/−10%), indicating that during PCR amplification the starting material was amplified by a factor of two during every cycle. Relative expression levels were analyzed using the 2^−ΔΔCt^ method [24], where ΔΔCt is the difference between the selected ΔCt value of a sample and the ΔCt of a control sample. Evaluation of 2^−ΔΔCt^ for the samples indicates the fold change in expression relative to the control.

### Data analyses not related to microarray experiments

Data in the figures are presented as mean ± S.E.M. of either three or four different experiments. Statistical analysis was performed using the two-sided unpaired *t*-test (GraphPad Prism 4, version 4.03). Differences were designated as indicated in the figures.

## Results

### Effect of testosterone on Oat1- and Oat3-promoter constructs in OK cells

**Figure 1:**
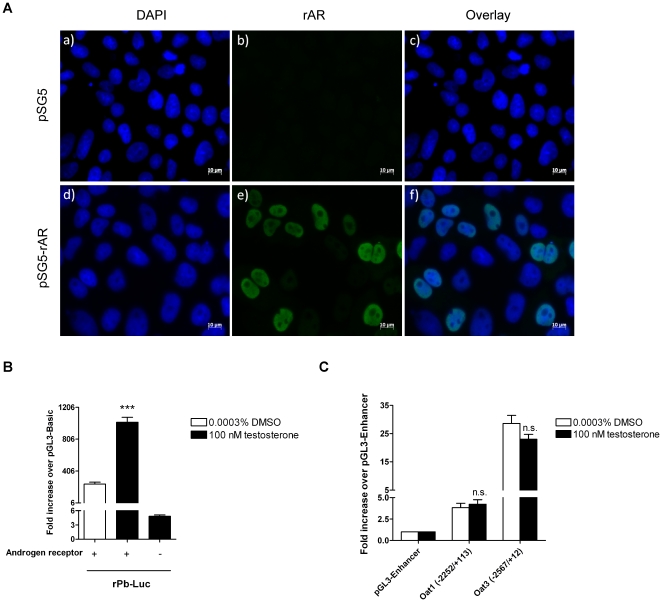
Effect of testosterone on probasin, Oat1- and Oat3-promoter activity. OK cells were either transfected with pSG5 or pSG5-rAR, and rAR expression and cellular localization were analyzed using immunofluorescence staining (green color rAR, blue color DAPI staining; excitation wavelength 488 nm and 365 nm) (1A). Promoter constructs of rat probasin, Oat1 and Oat3, and the expression vector for rAR were transiently transfected into OK cells (1B and 1C). Cells were cultured 43 h with either 100 nM testosterone (black bars) or with 0.0003% DMSO as control (white bars) (1B and 1C). Luciferase activity was measured and firefly luciferase was normalized to *Renilla* luciferase. Data are reported as the fold increase presented as mean ± S.E.M.; n = 4; n.s.: not significant; ***: p<0.001, significantly different from control (0.0003% DMSO).

*In silico* analysis of 3 kb upstream of the transcription start site of Oat1 and Oat3 promoters were investigated using MatInspector. In both promoters two putative androgen response elements (AREs) were postulated (Table 2), however none of which showed a perfectly conserved binding site (consensus sequence: GGTACAnnnTGTTCT) [25], [26]. The role of testosterone in the regulation of Oat1 and Oat3 promoter activity were investigated using luciferase assays as described in material and methods (Figure 1). Cellular localization of transfected rAR revealed an expression with an exclusive nuclear localization (Figure 1A). Transfection efficiency of rAR was 39.1%. The functional activity of rAR was confirmed by rat probasin promoter (rPb-Luc), used as positive control. rPb-Luc was significantly activated by 100 nM testosterone (Figure 1B). In the absence of rAR, rat probasin promoter was not enhanced by testosterone, demonstrating that OK cells do not express endogenous rAR (Figure 1B). The activation of rPb-Luc is regulated via two functional ARE (Table 2) [27]. No significant induction by 100 nM testosterone of Oat1- and as well as Oat3-promoter activities were observed (Figure 1C). The comparison of Oat1- and Oat3-promoter activity revealed a stronger basal promoter activity of Oat3 as compared to Oat1 (Figure 1C).

**Table 2:** *In silico* analyses of Oat1 and Oat3 promoter.

Promoter (3 kb)	Binding site	Sequence	Position
**Oat1**	ARE	5′-gcaggactcagtgttttgt-3′	−2006 to −1988
	ARE	3′-gtagccctggctgtcctgg-5′	−1655 to −1637
	BCL6	5′-tctttcctggtaagaga-3′	−2352 to −2336
	BCL6	5′-gagttccaggacagcca-3′	−1660 to −1644
	BCL6	5′-taatttttagaaataaa-3′	−1295 to −1279
	BCL6	5′-ctgttcctacaagttat-3′	−1143 to −1127
	BCL6	5′-tatttcccagagcccag-3	−492 to −476
**Oat3**	ARE	5′-ggctgcctacctgttctgt-3′	−2863 to −2845
	ARE	5′-gttgggctctgtgtactct-3′	−1923 to −1905
	BCL6	3′-accttccgtgaaaaaca-5′	−2055 to −2039
	BCL6	5′-gggtccctggaaatagt-3′	−1366 to −1350
	BCL6	5′-gacttcatagaaaactc-3′	−1174 to −1158
	BCL6	5′-atactcatagaaataaa-3′	−614 to −598
	BCL6	3′-aggttcgtggagaatgt-5′	−439 to −423
	BCL6	5′-cccttcccagattctct-3′	−279 to −263
**Probasin minimal promoter (−426 to +28 bp)**	ARE a)	5′-tgatagcatcttgttctta-3	−243 to −225
	ARE a)	5′-taggttcttggagtacttt-3′	−138 to −120
**consensus sequence:**	ARE	ggtacannntgttct [25], [26]	
**consensus sequence:**	BCL6	ttcct(a/c)gaa [28]	

Oat: organic anion transporter; ARE: androgen response element; BCL6: B-cell CLL/lymphoma 6;

n: any nucleotide, a) functional ARE published in [27].

### Sex-dependent expression of Oat1 and Oat3 in Han Wistar rats

**Figure 2:**
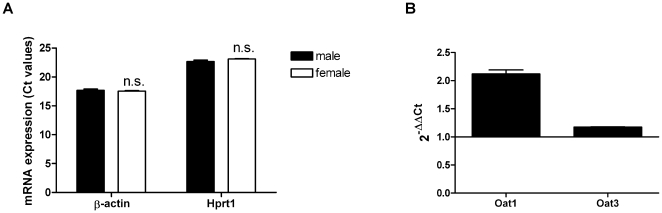
Sex-dependent expression of Oat1 and Oat3 in rat cortical kidney slices. Levels of β-actin, Hprt1, Oat1 and Oat3 were analyzed by TaqMan® real-time PCR in total RNA isolated from four male and four female cortical kidney slices. The mRNA expression of reference genes β-actin and Hprt1 were investigated by comparing their Ct values between male and female (2A). Levels of Oat1 and Oat3 were determined using 2^−ΔΔCt^ method, at which β-actin was the reference gene (2B). ΔΔCt values were calculated as ΔCt male - ΔCt female. n_male_ = 4; n_female_ = 4.

Based on TaqMan® real-time PCR we investigated the sex-dependent expression of Oat1 and Oat3 in cortical kidney slices from four male and four female rats. Since sex differences were investigated, two reference genes β-actin and Hprt were used. The mRNA levels of both reference genes were not altered under the experimental conditions (Figure 2A). TaqMan® real-time PCR revealed sex-dependent expression of Oat1 and Oat3 (Figure 2B). Thereby the sex-dependent difference of Oat1 between male and female was higher as compared to Oat3 (Figure 2B).

### Sex-dependent gene expression profiling

**Figure 3:**
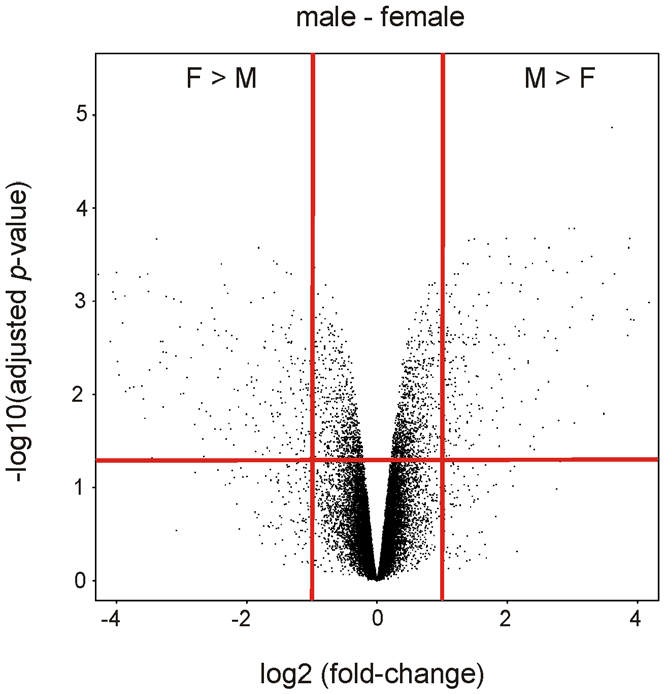
Volcano plot of microarray analysis. In this microarray a total of 22,863 probes were analyzed. On the y-axis the negative log10 of the adjusted *p*-value and on the x-axis the log2 of the fold-change is plotted. Each probe is represented as a dot. Low *p*-values (highly significant) are localized at the top of the plot. Probes that are expressed higher in females have a negative log2 fold-change appearing at the left side and probes that are expressed higher in males have a positive log2 fold-change appearing at the right side. The horizontal red line denotes the threshold for *p* = 0.05. The vertical red lines denote the two-fold thresholds.

**Figure 4:**
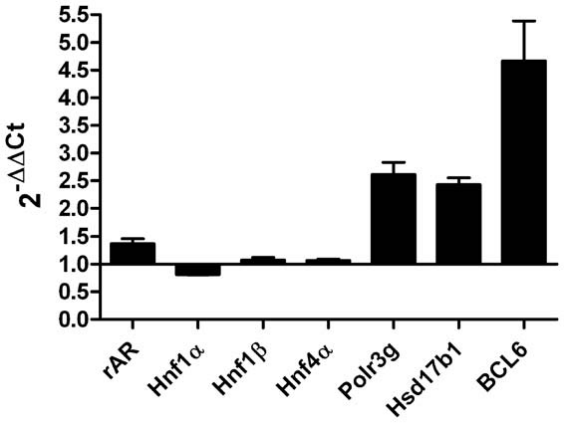
Verification of microarray results using TaqMan® real-time PCR. Gene expressions were verified by TaqMan® real-time PCR in total RNA isolated from four male and four female cortical kidney slices. Levels of all genes were determined using 2^−ΔΔCt^ method, at which β-actin was the reference gene. ΔΔCt values were calculated as ΔCt male - ΔCt female. n_male_ = 4; n_female_ = 4.

The gene expression profiles of cortical kidney slices from the four male and four female rats used previously, were analyzed using the SurePrint G3 Rat GE 8×60K Microarray Kit from Agilent Technologies. The volcano plot gives a first overview of gene expression between males and females (Figure 3). Out of 22,863 investigated probes, 572 exhibit significant [−log10(adjusted *p*-value)<0.05] sex-dependent expression (log2 fold-change≤−1 or ≥1) (Figure 3). 293 probes were higher expressed in females (log2 fold-change≤−1), and 281 probes were higher expressed in males (log2 fold-change≥1) (Figure 3). After exclusion of probes with a FDR >5% and probes without Gene ID, 160 genes were higher expressed in females and 175 genes higher in males (Table S1). The alignment with the “Rat Proximal Tubule Transcriptome Database” and the removal of duplicated genes revealed 56 genes with significant sex-dependent expression in proximal tubule cells (Table 3). Out of these 56 genes, 13 genes showed a higher expression in females whereas 43 genes showed a higher expression in males. Genes were classified into different groups according to their function included: twenty-one enzymes (18×M>F, 3×F>M), nine membrane proteins and receptors (7×M>F, 2×F>M), two transcription factors (1×M>F, 1×F>M), six transport proteins (4×M>F, 2×F>M), six signal transduction (4×M>F, 2×F>M) and twelve other genes (9×M>F, 3×F>M) (Table 3). TaqMan® real-time PCR was used to verify selected microarray results. Seven genes were chosen as a result of their possible involvement in the transcriptional regulation of the sex-dependently expressed Oat1 and Oat3: Hnf1α, Hnf1β, Hnf4α, rAR (GSE34565), Hsd17b1, BCL6, and Polr3g (bold letters in Table 3). All three validated hepatocyte nuclear factors (Hnf1α, Hnf1β, and Hnf4α) showed no sex-dependent expression (Figure 4), confirming the microarray results (GSE34565). Validation of rAR showed a significant male-dominant expression (Figure 4). This result did not coincide with that indicated by microarray where rAR showed no sex-dependent expression (GSE34565). The levels of Polr3g, Hsd17b1, and BCL6 were clearly higher in males as compared to females (Figure 4), confirming the microarray results (Table 3).

**Table 3:** Sex-dependently expressed genes in rat proximal tubule cells.

Symbol	Description	log2 FC (m- f)	P. Value (m-f)	FDR (m-f)
	**ENZYMES**			
Aldh1a1	aldehyde dehydrogenase 1 family, member A1	1,01	3,85E-06	0,09%
Baat	bile acid Coenzyme A: amino acid N-acyltransferase (glycine N-choloyltransferase) choloyltransferase)chcchcholoyltransferasecholoyltransferasecholoyltransferase)	−1,24	1,22E-03	2,40%
Cth	cystathionase (cystathionine gamma-lyase)	−1,01	1,24E-04	0,61%
Cyp2d4v1	cytochrome P450, family 2, subfamily d, polypeptide 4	1,11	4,39E-06	0,10%
Cyp4a2	cytochrome P450, family 4, subfamily a, polypeptide 2	3,03	9,25E-04	2,04%
Ddx19a	DEAD (Asp-Glu-Ala-Asp) box polypeptide 19a	1,05	6,57E-05	0,43%
Dnm3	dynamin 3	2,64	2,16E-07	0,03%
Eepd1	endonuclease/exonuclease/phosphatase family domain containing 1	1,02	2,67E-04	0,98%
Es22	esterase 22	2,02	3,16E-07	0,03%
F3	coagulation factor III (thromboplastin, tissue factor)	1,10	2,80E-05	0,27%
Hao1	hydroxyacid oxidase (glycolate oxidase) 1	1,88	1,24E-04	0,61%
Hmgcs2	3-hydroxy-3-methylglutaryl-Coenzyme A synthase 2 (mitochondrial)	3,30	9,19E-06	0,16%
**Hsd17b1**	**hydroxysteroid (17-beta) dehydrogenase 1**	**1,52**	**1,93E-05**	**0,23%**
Oat	ornithine aminotransferase (gyrate atrophy)	−1,36	3,03E-05	0,28%
Pecr	peroxisomal trans-2-enoyl-CoA reductase	1,15	9,75E-05	0,53%
Proc	protein C	1,01	2,22E-07	0,03%
Ptgds	prostaglandin D2 synthase (brain)	2,19	2,46E-06	0,08%
Rasa2	RAS p21 protein activator 2	1,04	5,92E-05	0,41%
Rasl12	RAS-like, family 12	2,18	2,12E-05	0,24%
Rdh2	retinol dehydrogenase 2	1,56	4,49E-04	1,31%
Usp9x	ubiquitin specific peptidase 9, X-linked	2,35	2,53E-03	3,60%
	**MEMBRANE PROTEINS/RECEPTORS**			
Asgr1	asialoglycoprotein receptor 1	1,49	3,35E-05	0,29%
B4galt5	UDP-Gal:betaGlcNAc beta 1,4-galactosyltransferase, polypeptide 5	1,00	1,23E-05	0,18%
Cd1d1	CD1d1 molecule	−1,14	7,03E-04	1,74%
Kifc1	kinesin family member C1	1,37	3,41E-05	0,29%
Prlr	prolactin receptor	1,14	1,82E-04	0,78%
Sectm1b	secreted and transmembrane 1B	1,21	5,43E-05	0,40%
Stra6	stimulated by retinoic acid gene 6	−1,16	9,07E-06	0,16%
Tmem144	transmembrane protein 144	1,02	3,89E-05	0,32%
Trim59	tripartite motif-containing 59	1,02	5,78E-06	0,12%
	**TRANSCRIPTION FACTORS**			
Arid3b	AT rich interactive domain 3B (Bright like)	−1,48	1,65E-04	0,73%
**Bcl6**	**B-cell CLL/lymphoma 6**	**2,18**	**1,61E-05**	**0,21%**
	**TRANSPORT PROTEINS**			
Abca7	ATP-binding cassette, sub-family A (ABC1), member 7	−1,35	1,40E-05	0,19%
Abcb1b	ATP-binding cassette, sub-family B (MDR/TAP), member 1B	−2,66	3,96E-03	4,65%
Ostalpha	organic solute transporter alpha	1,13	3,06E-05	0,28%
Slc10a1	solute carrier family 10 (sodium/bile acid cotransporter family), member 1	1,12	3,98E-04	1,21%
Slc30a2	solute carrier family 30 (zinc transporter), member 2	1,26	7,12E-06	0,13%
Ust5r	integral membrane transport protein UST5r	1,07	1,85E-04	0,79%
	**SIGNAL TRANSDUCTION**			
Cklf	chemokine-like factor	−1,03	6,79E-05	0,44%
Cks2	CDC28 protein kinase regulatory subunit 2	1,06	3,81E-03	4,55%
Dock5	dedicator of cytokinesis 5	−1,58	1,75E-03	2,93%
Fgf13	fibroblast growth factor 13	1,12	3,32E-06	0,09%
Gas2	growth arrest-specific 2	1,40	5,73E-07	0,04%
Nrep	neuronal regeneration related protein	1,16	2,97E-04	1,02%
	**OTHERS**			
Cldn1	claudin 1	−1,54	5,66E-07	0,04%
Ddit4l	DNA-damage-inducible transcript 4-like	−1,76	3,62E-03	4,40%
Espn	espin	1,21	1,31E-03	2,51%
Klhl14	kelch-like 14 (Drosophila)	−1,31	2,68E-05	0,27%
Mlph	melanophilin	1,91	1,53E-06	0,06%
Obfc2a	oligonucleotide/oligosaccharide-binding fold containing 2A	1,17	2,45E-05	0,26%
Picalm	phosphatidylinositol binding clathrin assembly protein	1,14	2,96E-06	0,08%
**Polr3g**	**polymerase (RNA) III (DNA directed) polypeptide G (32 kD)**	**1,34**	**3,14E-05**	**0,28%**
Rpp25	ribonuclease P 25 subunit (human)	1,77	4,64E-05	0,35%
Rufy3	RUN and FYVE domain containing 3	1,06	2,06E-03	3,20%
Spc25	SPC25, NDC80 kinetochore complex component, homolog (S. cerevisiae)	1,02	1,73E-03	2,92%
Tox	thymocyte selection-associated high mobility group box	1,07	1,81E-05	0,22%

FC: log2 fold-change (FC)≤−1 or ≥1; FDR: false discovery rate <5%; P. value: p<0.05; (m-f): male-female. Genes are grouped, and displayed within these groups in an alphabetical order. Genes shown with a negative log2 FC are higher expressed in females while genes with a positive log2 FC are higher expressed in males. The expression of all genes was aligned with (http://dir.nhlbi.nih.gov/papers/lkem/pttr/). Bold marked genes were selected for verification by TaqMan® real-time PCR.

### *In silico* analyses of potential BCL6 binding sites in the Oat1- and Oat3-promoter region

In the analyzed Oat1-promoter there are five potential BCL6 binding sites postulated none of which was a perfectly conserved binding site (consensus sequence: TTCCT(A/C)GAA) [28] (Table 2). *In silico* analysis of the Oat3-promoter revealed six potential BCL6 binding sites, of which none did correlate completely with the consensus sequence (Table 2). The promoter construct Oat1 (−1266/+113) included two predicted BCL6 binding sites, the Oat1 (−1666/+113) construct included four predicted BCL6 binding sites and the Oat1 (−2252/+113) included four predicted BCL6 binding sites (Table 2).

The shortest promoter construct Oat3 (−444/+12) has two predicted BCL6 binding sites (Table 2). In the Oat3 promoter construct (−752/+12), three BCL6 responsive elements are located. The longest Oat3 promoter construct (−2567/+12) included six BCL6 binding sites (Table 2).

### Activation of Oat1- and Oat3-promoter by BCL6

**Figure 5:**
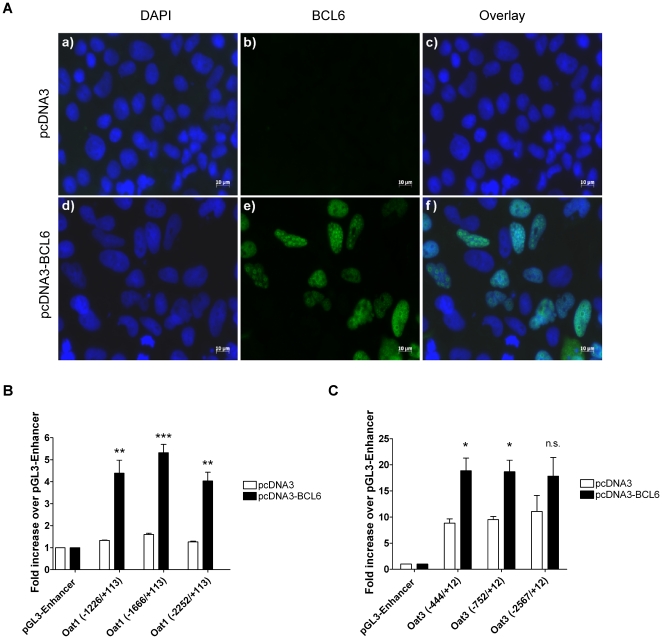
Effects of BCL6 on Oat1- and Oat3-promoter activity. OK cells were either transfected with pcDNA3 or pcDNA3-BCL6, and BCL6 expression and cellular localization were analyzed using immunofluorescence staining (green color BCL6, blue color DAPI staining; excitation wavelength 488 nm and 365 nm) (5A). Promoter constructs of Oat1 and Oat3, and the expression vector for BCL6 were transiently transfected into OK cells (5B and 5C). Luciferase activity was measured and firefly luciferase was normalized to *Renilla* luciferase. Data are reported as the fold increase over pGL3-Enhancer and presented as mean ± S.E.M.; n = 3; n.s.: not significant; *: p<0.05; **: p<0.01; ***: p<0.001, significantly different from control (pcDNA3).

As a positive control for transfection and expression in OK cells, BCL6 expression was investigated using immunofluorescence (Figure 5A). Characterization of BCL6 cellular localization revealed an exclusive nuclear localization (Figure 5A). Transfection efficiency of BCL6 was 23,3%. To test for a possible influence of BCL6 on the promoters of Oat1 and Oat3, OK cells were transfected with different promoter constructs, pcDNA3-BCL6 or empty pcDNA3 vector, and their luciferase activity were measured (Figure 5B and 5C). All three Oat1-promoter constructs showed a significant activation by BCL6 compared to the control (Figure 5B). The promoter constructs Oat3 (−444/+12) and (−752/+12) were activated by BCL6 (Figure 5C). The promoter activity of the longest Oat3 construct (−2567/+12) was not significantly altered but showed a positive trend for an activation by BCL6 (Figure 5C).

## Discussion

The aim of this study was to identify sex-dependently expressed genes in the rat proximal tubule cells that may be involved in the transcriptional regulation of Oat1 and Oat3. Since 1955 it is known that the uptake of the typical Oat1 substrate, *p*-aminohippurate (PAH), by rat renal cortical slices is higher in males compared to females [29]. Testosterone stimulated the uptake [30] possibly by having a positive influence on the expression of functional transporting proteins [31]. PAH is absorbed from the blood into proximal tubule cells by Oat1 and Oat3 in rat kidneys [32]–[36] and, in rat renal proximal tubule cells, a higher expression of Oat1 and Oat3 in males compared to females has been reported [19], [37]. Moreover, it has been shown that Oat1 and Oat3 expression is increased by testosterone [19]. Two different AREs were predicted in the promoter of Oat1 and Oat3 in our *in silico* analysis. Surprisingly, testosterone showed no stimulating effect on the promoter activity of Oat1 and Oat3, although the minimal promoter of rat probasin (rPb-Luc) was activated under the same conditions. These results suggest that the predicted AREs in the promoter of Oat1 and Oat3 are not functional, and that these transport proteins are not directly activated by the classical androgen receptor mediated transcriptional pathway. Given the known sex-dependent expression of Oat1 and Oat3 in rat proximal tubule cells [19], [37], we concluded that hitherto unknown factors are involved in the transcriptional male-dominant expression of Oat1 and Oat3.

Using microarray analysis, we examined the expression of 17,406 different genes and found that only 56 genes were significantly sex-dependently expressed. This relatively small number of sex-dependently expressed genes is due to the fact that we only analyzed genes that are localized within the proximal tubule cells. The promising Oat1 and Oat3 regulators, Hnf1α, Hnf1β, and Hnf4α are expressed in the proximal tubule cells (http://dir.nhlbi.nih.gov/papers/lkem/pttr/), but revealed no sex-dependent expression in our microarray analysis and real-time PCR. For human OAT1-promoter the activation by HNF4α was demonstrated [38]. The promoters of mouse Oat1, human OAT1 and OAT3 are activated by HNF1α and HNF1β [39], [40].

The possible Oat1 and Oat3 regulator, rat androgen receptor (rAR) that is expressed in the proximal tubule cells (http://dir.nhlbi.nih.gov/papers/lkem/pttr/) showed no sex-dependent expression in the microarray, but demonstrated a significant male-dominant expression by real-time PCR. The decreased sensitivity of microarrays in comparison to real-time PCR may explain the different results. It is known that both real-time PCR and microarray analysis have inherent pitfalls that could influence the data obtained from each method, resulting in disagreement [41]. The false discovery rate (FDR) of rAR was about 24%, indicating a false positive microarray result that was verified by real-time PCR revealing a significant male-dominant expression. Despite the higher expression of rAR in the male kidneys, rAR is probably not a direct regulator of Oat1 and Oat3 promoters, as we could demonstrate in our study.

Out of the 56 sex-dependently expressed genes three genes that showed a male-dominant sex-dependent expression in the microarray, Polr3g, Hsd17b1, and BCL6 were selected for verification. The real-time PCR analysis clearly confirmed the microarray results for these three genes.

The first validated gene was Polr3g that is a subunit of the dissociable RNA polymerase (pol) III subcomplex [42]. Pol III transcribes especially short non-coding RNAs, for example tRNA, 5S RNA, 7SK RNA, U6 small nuclear RNA and H1 RNA that are involved in processing of pre-rRNA, mRNA and tRNA [42], [43]. This subcomplex is responsible for the initiation of Pol III transcription [42]. This so far unknown male-dominant Polr3g expression could enhance the initiation of pol III transcription, leading possibly to a rather indirect effect on the expression of Oat1 and Oat3.

Hsd17b1 was the second validated gene and represents a promising candidate gene for sex-dependent Oat1 and Oat3 regulation. Members of the 17β-hydroxysteroid dehydrogenase (HSDs) family play an important role in the estrogen and androgen steroid biosynthesis [44]–[46]. Human HSD17b1 selectively converts estrone to estradiol [44]. The rat Hsd17b1 mediates the reduction of androstenedione to testosterone as efficiently as the analogous reduction from estrone to estradiol [47]. The male-dominant renal Hsd17b1 expression could be responsible for an increased testosterone concentration in proximal tubule cells. The increase of Oat1 and Oat3 protein expression by testosterone was published [19], but this increase is potentially not mediated by the classical androgen receptor mediated transcriptional pathway as demonstrated in our contribution. Possibly, the elevated testosterone concentration could activate a, till now unknown, transcription factor, which then activates Oat1 and Oat3 expression.

The third real-time PCR validated gene was the transcription factor BCL6, that was identified and characterized in B-cells [48]. BCL6 is necessary during embryonic development, plays a role in germinal center formation, and is essential in the immune response [28], [48]. BCL6 has also been reported to be a proto-oncogen, whereby the intact coding sequence of BCL6 is often translocated, obtaining a new changed 5′ non-coding promoter region [28]. In many cases BCL6 acts as a transcriptional repressor due to its ability to recruit the known transcriptional repressors nuclear receptor corepressor (N-CoR), silencing mediator of retinoid and thyroid receptor (SMRT), and BCL6 interacting corepressor (BCoR) [28], [48]. These three transcriptional repressors recruit histone deacetylases 1 and 2 [48]. BCL6 inhibits the expression of different genes like p21 and cyclin D2 [28], [48]. Moreover it could repress the activity of NFκB by a direct protein-protein interaction between BCL6 and the subunits of NFκB [49]. BCL6 could also act as an activator leading to alteration in the growth cycle of the cell [50]. In our study, a considerably higher BCL6 expression in males compared to females was found. A comparable male-dominant expression of BCL6 has been demonstrated in the rat liver, whereas a possible role of BCL6 in the regulation of Oats has not been investigated [51]. Although all three validated genes are promising candidate genes for sex-dependent regulation of Oat1 and Oat3, we decided to focus on the possible involvement of BCL6 as a result of predicted BCL6 binding sites in the Oat1 and Oat3 promoter region.

BCL6 is known to bind to a specific DNA sequence with a core sequence of TTCCT(A/C)GAA [28]. Luciferase activity assays revealed a significant activation of Oat1 as well as Oat3 promoter by BCL6. All three Oat1 as well as Oat3 promoter constructs contain predicted BCL6 binding sites. Regarding the amplitude of fold induction, all Oat1 constructs were activated to a comparable amount, indicating that the first and/or second BCL6 binding site is may be the critical site for activation of Oat1 promoter constructs by BCL6. Oat3 promoter constructs were also activated to a comparable extent by BCL6 and possibly directly regulated via first and/or second BCL6 binding site. Our results implicate that male-dominant BCL6 is a promising transcription factor in the promoter activation of sex-dependently expressed Oat1 and Oat3. Interestingly, BCL6 is able to repress the activity of NFκB at the post-transcriptional level due to protein-protein interaction [49]. In renal ischemia the expression of rat renal Oat1 and Oat3 was decreased [52], and that of NFκB was increased [53]. Therefore it is possible that male-dominant BCL6 expression suppresses the activity of NFκB, leading indirectly to the higher expression of Oat1 and Oat3 in males. NFκB itself is expressed in the proximal tubule cells (http://dir.nhlbi.nih.gov/papers/lkem/pttr/) but exhibited no sex-dependent expression in our microarray analysis (GSE34565).

This study presents a first description of sex-dependent gene expression in the kidneys and especially in the proximal tubule cells (GSE34565). The sex differences were identified in several gene groups, e.g. enzymes, membrane proteins and receptors, transcription factors, transport proteins, genes involved in the signal transduction pathways, and others. Some candidate genes like Polr3g, Hsd17b1 and BCL6 might be directly or indirectly involved in sex-dependent expression of Oat1 and Oat3. Our results showed that BCL6 activated the promoters of Oat1 and Oat3 and constituted a promising candidate gene for their sex-dependent regulation. To further elucidate the involvement of BCL6 in the sex-dependent expression of Oat1 and Oat3, additional experiments are required.

## Supporting Information

Table S1 **Sex-dependently expressed genes in rat cortical kidney slices.** FC: log2 fold-change (FC)≤−1 or ≥1, FDR: false discovery rate, (m-f): male-female. Genes are displayed in an alphabetical order, starting with negative log2 FC representing genes higher expressed in females, followed by genes with a positive log2 FC representing higher expression in males. Probes with a FDR >5% and probes with no Gene ID are excluded in this list.
(XLS)
